# P147: Assessing the thoroughness of hand hygiene: “To see is to believe”

**DOI:** 10.1186/2047-2994-2-S1-P147

**Published:** 2013-06-20

**Authors:** Y-C Chen, K-L Tien, E Chen, S-C Pan, I-C Hung, W-H Sheng, S-C Chang

**Affiliations:** 1 Center for Infection Control, Department of Internal Medicine, Taipei, Taiwan, Province of China; 2 Center for Infection Control, National Taiwan University Hospital and College of Medicine, Taipei, Taiwan, Province of China; 3 Taipei American School, Taipei, Taiwan, Province of China; 4 Department of Internal Medicine, National Taiwan University Hospital and College of Medicine, Taipei, Taiwan, Province of China

## Introduction

Many staff challenged infection control personnel (ICP) regarding the rationale of hand rubbing time. Furthermore, few if any recommendation described how to evaluate correct hand hygiene (HH) technique. Here we descried the impact of a stress-free, “to see is to believe” program on proper HH technique conduced in May 2012 at a 2200-bed teaching hospital in Taiwan.

## Objectives

Adapting a physical method to evaluate the thoroughness of HH

## Methods

The staff volunteered to sign up for the campaign. The thoroughness of HH was evaluated by physical method. Staff rubbed their hands with a fluorescent substance as they would normally do with alcohol-based hand rub, and placed their hands under an ultraviolet light box to identify any areas they might have missed. Two ICP administered the test and assessed each person’s performance and recorded on a graph for residue points and location (37 parts of the hands). We also recorded the time of hand rubbing. The results were recorded anonymously. Six months later, ICPs conducted hospital-wide survey by direct observation of HH compliance and technique.

## Results

Among 85 wards, 388 staff from 30 wards participated in this study. The hand rubbing time for all participants were more than the recommended 10-15 seconds with an average of 57±26.4 seconds. The hand rubbing time was not affected by age, gender, and professional categories. 45.2% have zero residuals. 74.7% had less than 3 residue points. The average residue point is 2±2.8 points. There is no correlations among hand rubbing time, participants’ preceded confidence, and the residue points. We found that participants who have damaged skin had more residues. The highest percentage of the residue points lie in the tips of the nails (38.6%, 340/880), followed by figure tips (17.4%). Follow-up survey showed the proportion of staff with correct HH technique increased from 76.6% in 2011 to 81.3% in 2012. The composite compliance rate increased as well (from 82.7% to 85.5%).

## Conclusion

We adapted a physical method to evaluate the thoroughness of HH and discovered the parts of the hands that are often neglected during HH.

## Disclosure of interest

None declared

